# On Four-Rich Points Defined by Pencils

**DOI:** 10.1007/s00454-026-00884-0

**Published:** 2026-09-19

**Authors:** Michalis Kokkinos, Audie Warren

**Affiliations:** 1 Institute for Algebra, Johannes Kepler University, Linz, Austria; 2 Johann Radon Institute for Computational and Applied Mathematics (RICAM), Austrian Academy of Sciences, Linz, Austria

**Keywords:** Incidences, Rich-points, Sheaves, 52C10 (primary), 05B30 (secondary)

## Abstract

In this paper we study the number of four-rich points defined by pencils of certain algebraic objects. Our main result concerns the number of four-rich points defined by four sheaves of planes; under certain non-degeneracy conditions, we prove that four sheaves of *n* planes in ${\mathbb {P}}^3$ determine at most $O(n^{8/3})$ four-rich points. We prove this using the four dimensional Elekes-Szabó theorem. Using the same method, we prove an upper bound on the number of four-rich points determined by four sets of concentric spheres in $\mathbb {C}^3$. Furthermore, using the same technique with the 3-d Elekes-Szabó theorem, one can prove upper bounds on four-rich points determined by various configurations of lines/circles in the plane $\mathbb {R}^2$; we give one such example, involving two pencils of lines and two pencils of concentric circles in $\mathbb {R}^2$

## Introduction

In this paper, we study the intersection properties of certain families of objects. Specifically, we give various upper bounds for the number of four-rich points defined by finite subsets of one-dimensional families of algebraic objects. An example of such a result is given in [8], where the authors show that given four sets of concurrent lines in ${\mathbb {R}}^2$, where each set has size *n* and the centers are not all collinear, the number of four-rich points is of size $O(n^{11/6})$. The concept here is that apart from some degenerate situations (e.g. collinear/coplanar points), such configurations cannot define many four-rich points. Our main result concerns the three dimensional version of this problem; we show that four *sheaves of* *n* *planes* in complex projective space ${\mathbb {P}}^3$ define few four rich points, under certain non-degeneracy conditions. We recall that a sheaf of planes is a set of planes which all contain a common line, and we call this common line the *axial line* of the sheaf. If the four axial lines of the sheaves are pairwise skew, and the sheaves do not all contain a common transversal line of all axial lines, we prove that we do indeed get few four-rich points. In Sect. 3.1 we give a construction to show that such restrictions are necessary.

### Theorem 1

Let $\Pi _1,\Pi _2,\Pi _3,\Pi _4$ be four sheaves of planes in ${\mathbb {P}}^3$, each of size *n*. Suppose that the axial lines of the sheaves are pairwise skew, and that no transversal line of the axial lines is contained in a plane from each sheaf. Then the number of four rich points determined by these sheaves is $O(n^{8/3})$.

Secondly, we consider the number of four-rich points which can be defined by four sets of concentric spheres in ${\mathbb {C}}^3$.

### Theorem 2

Let ${\mathcal {S}}_1, {\mathcal {S}}_2, {\mathcal {S}}_3, {\mathcal {S}}_4$ be four sets of *n* concentric spheres in ${\mathbb {C}}^3$, whose centres do not lie on a common plane, and no three centres are collinear. Then the number of four rich points determined by these four sets is $O(n^{8/3})$.

As a corollary of Theorem 2, we obtain the following Erdős pinned distances type result. In the following, we define *D*(*q*, *P*) for $q\in {\mathbb {R}}^3$ and $P \subseteq {\mathbb {R}}^3$ to be the set of distances defined between *q* and *P*.

### Corollary 1

Let $P \subseteq {\mathbb {R}}^3$ be a finite set of points, and let $q_1,q_2,q_3,q_4 \in {\mathbb {R}}^3$ be four non-coplanar points, with no three collinear. Then we have

$$\max _{i} D(q_i,P) \gg |P|^{3/8}.$$

Note that the trivial lower bound is $|P|^{1/3}$, which is tight if three of the points are collinear. This is shown by a simple construction given in Sect. 4.1.

In the last section, we give an upper bound on the number of four-rich points defined by two pencils of lines and two pencils of concentric circles in $\mathbb {R}^2$. Research in this direction was initiated by the seminal paper of Elekes and Szabó [4]. Their main result, listed as Theorem 3 below, was applied to derive an upper bound for the number of intersection points between circles belonging to three families of concentric circles, see [4, Theorem 32]. Parallel to Theorem 2 and Corollary 1, that result can also be seen as a bound on the number of distinct distances between a finite point set in the plane and three other non collinear points. We prove that for two pencils of lines and two pencils of concentric circles of size *n*, the number of four-rich points is $O(n^{12/7})$. We defer the statement of this result to the last section.

### The Elekes-Szabó Problem

The Elekes-Szabó problem [4] concerns itself with the number of intersection points between an algebraic surface and a finite Cartesian product. The three-dimensional version, concerning the number of intersections between the zero set of a polynomial *F*(*x*, *y*, *z*) and a finite set of the form $A \times B \times C$, has seen a wide range of applications to combinatorial problems, see for instance [7, 9] and the survey paper [2]. We state the most recent version of the Elekes-Szabo theorem due to Solymosi and Zahl [10], which builds upon the work of Raz, Sharir, de Zeeuw [5].

#### Theorem 3

(Solymosi, Zahl [10]) Let $F\in \mathbb {C}[x,y,z]$ be a degree *d* irreducible polynomial with each of $F_x,F_y,F_z$ not identically zero. Then one of the two following statements hold.

- For all finite sets $A,B,C\subseteq \mathbb {R}$ with |A|=|B|=|C|=n, we have
  $$\begin{aligned} |V(F)\cap A\times B\times C|\ll _{d}n^{12/7}. \end{aligned}$$
- There exists a one-dimensional subvariety $Z_0\subseteq Z(F),$ such that for every $p\in Z(F)\backslash Z_0$, there are open sets $D_1, D_2,D_3$ with $p \in D_1 \times D_2 \times D_3$ and bijective analytic functions $\phi _i:D_i\rightarrow \mathbb {C},$ with analytic inverses, such that for every $(x,y,z)\in D_1\times D_2\times D_3$ we have
  $$\begin{aligned} (x,y,z)\in Z(F)\quad \text {if and only if}\quad \phi _1(x)+\phi _2(y)+\phi _3(z)=0. \end{aligned}$$

A polynomial *F* which satisfies item two of Theorem 3 will be called *degenerate*. In order to apply Theorem 3 we need a way to test whether polynomials are degenerate. This is provided by the following derivative test. This result can be found in various places in the literature, for instance [9, Lemma 6], [4, Lemma 33], or the survey [2]. We state the variant over the complex numbers which uses rational functions, since this is all we will need in our application.

#### Lemma 1

(Degeneracy Test) Suppose that $F(x,y,z) \in {\mathbb {C}}[x,y,z]$ is a degenerate polynomial. Let *f*(*x*, *y*) be a rational function with $f_x$ and $f_y$ not identically zero, such that wherever *f* is defined, we have

z=f(x,y)⟺F(x,y,z)=0.

Then there exists a non-empty open set $U \subseteq \mathbb {C}^2$ such that

$$\frac{\partial ^2 (\ln |f_x/f_y|)}{\partial x \partial y}$$

is identically zero on *U*.

We prove Theorems 1 and 2 by using the four-dimensional Elekes-Szabo result of Raz, Sharir, and de Zeeuw [6], given as Theorem 4 below.

#### Theorem 4

(Raz, Sharir, de Zeeuw [6]) Let $F \in {\mathbb {C}}[x,y,z,w]$ be an irreducible polynomial of constant degree, with no partial derivative identically zero. Then one of the following two statements holds.

1. For any finite sets $A,B,C,D \subseteq {\mathbb {C}}$, we have
  $$\begin{aligned} |Z(F) \cap A \times B \times C \times D| \ll (|A||B||C||D|)^{2/3}+|A||B|+|A||C|\\+|A||D|+|B||C|+|B||D|+|C||D|. \end{aligned}$$
2. There exists a two-dimensional subvariety $Z_0 \subseteq Z(F)$ such that for all $p \in Z(F) \setminus Z_0$, there exist open sets $D_i \subseteq {\mathbb {C}}$ and bijective analytic functions $\phi _i: D_i \rightarrow {\mathbb {C}}$ with analytic inverses, for i=1,2,3,4, such that $p \in D_1 \times D_2 \times D_3 \times D_4$, and for all $(x,y,z,w) \in D_1 \times D_2 \times D_3 \times D_4$ we have
  $$F(x,y,z,w) = 0 \iff \phi _1(x) + \phi _2(y) + \phi _3(z) + \phi _4(w) =0.$$

Informally, the second case of Theorem 4 means that (locally) the zero set of *F* can be represented by a sum of four univariate functions. If this case occurs, we again call the polynomial *degenerate*. In order to use Theorem 4, we begin by proving a Lemma which gives a sufficient condition for a polynomial to be non-degenerate. It is of note that this upcoming degeneracy test appears to be simpler than the analogue Lemma 1.

## A 4-d Elekes-Szabó Degeneracy Test

In this section we prove the following degeneracy test lemma. The proof is analogous to the three-dimensional case, see for instance [9]. Note that this result is stated in contrapositive form to Lemma 1—this is because we will use it in this form to prove non-degeneracy of polynomials *F*(*x*, *y*, *z*, *w*).

### Lemma 2

Let $F(x,y,z,w) \in {\mathbb {C}}[x,y,z,w]$ be an irreducible polynomial with no partial derivative identically zero, such that we may rearrange the equation F(x,y,z,w)=0 to the form w=f(x,y,z) with *f* rational. If any of the expressions

$$\frac{\partial }{\partial z}\left( \frac{f_x}{f_y}\right) , \quad \frac{\partial }{\partial x}\left( \frac{f_y}{f_z}\right) , \quad \frac{\partial }{\partial y}\left( \frac{f_x}{f_z}\right) $$

do not vanish on any non-empty open set $U \subseteq \mathbb {C}^3$, then *F*(*x*, *y*, *z*, *w*) is non-degenerate.

### Proof

We prove this via contradiction. Suppose that *F*(*x*, *y*, *z*, *w*) is a degenerate polynomial, and let $U \subseteq \mathbb {C}^3$ be the Zariski open set where *f*(*x*, *y*, *z*) is defined. Then for some fixed point *p* in the set $\{(x,y,z,w) \in Z(F) \setminus Z_0: (x,y,z) \in U\}$, there exist (non-empty analytic) open sets $D_1,D_2,D_3,D_4 \subseteq {\mathbb {C}}$ such that $p \in D_1 \times D_2 \times D_3 \times D_4$, chosen small enough so that $D_1 \times D_2 \times D_3 \subseteq U$, and analytic bijective functions with analytic inverses $\phi _i: D_i \rightarrow {\mathbb {C}}$, i=1,2,3,4, such that on $D_1 \times D_2 \times D_3 \times D_4$, we have

$$F(x,y,z,w) = 0 \iff \phi _1(x) + \phi _2(y) + \phi _3(z) + \phi _4(w) = 0 \iff w = f(x,y,z).$$

Defining $\psi (t) = \phi _4^{-1}(-t)$, we see that we have $f(x,y,z) = \psi ( \phi _1(x) + \phi _2(y) + \phi _3(z))$ on the open set $D_1 \times D_2 \times D_3$. We then have

$$\begin{aligned} f_x&= \phi _1'(x)\psi '(\phi _1(x) + \phi _2(y) + \phi _3(z)) \\ f_y&= \phi _2'(y)\psi '(\phi _1(x) + \phi _2(y) + \phi _3(z)) \\ f_z&= \phi _3'(z)\psi '(\phi _1(x) + \phi _2(y) + \phi _3(z)). \end{aligned}$$

Therefore we have

$$\frac{f_x}{f_y} = \frac{\phi _1'(x)}{\phi _2'(y)}, \quad \frac{f_y}{f_z} = \frac{\phi _2'(y)}{\phi _3'(z)}, \quad \frac{f_x}{f_z} = \frac{\phi _1'(x)}{\phi _3'(z)}$$

from which we see that on the open set $(D_1 \times D_2 \times D_3) \setminus (V(f_y) \cup V(f_z)) \subseteq U$, (which is not empty since $V(f_y)$ and $V(f_z)$ are Zariski open subsets of hypersurfaces in $\mathbb {C}^3$ since *F* has non-vanishing partial derivatives), we must have

$$\begin{aligned} \frac{\partial }{\partial z} \left( \frac{f_x}{f_y} \right) = \frac{\partial }{\partial x} \left( \frac{f_y}{f_z} \right) = \frac{\partial }{\partial y} \left( \frac{f_x}{f_z} \right) = 0. \end{aligned}$$

The lemma then follows. □

In the appendix we prove a short Mathematica package which can be used to apply this degeneracy test. In all of our applications of the 4-d degeneracy test, we have applied this code.

## Rich Points from Sheaves of Planes

### Construction

Before giving the proof of Theorem 1, we give a construction showing that certain sets of sheaves of planes can give infinitely many four-rich points - this is important context for the proof of Theorem 1. Note that in the following construction, the four lines can be *any* four pairwise skew lines - we show that there always exists a transversal line which must be excluded from the sheaves.

#### Construction 1

There exist four sheaves of planes $\Pi _1,\Pi _2,\Pi _3,\Pi _4$, each of size *n*, whose axial lines are pairwise skew, but define infinitely many four-rich points.

**Fig. 1:**
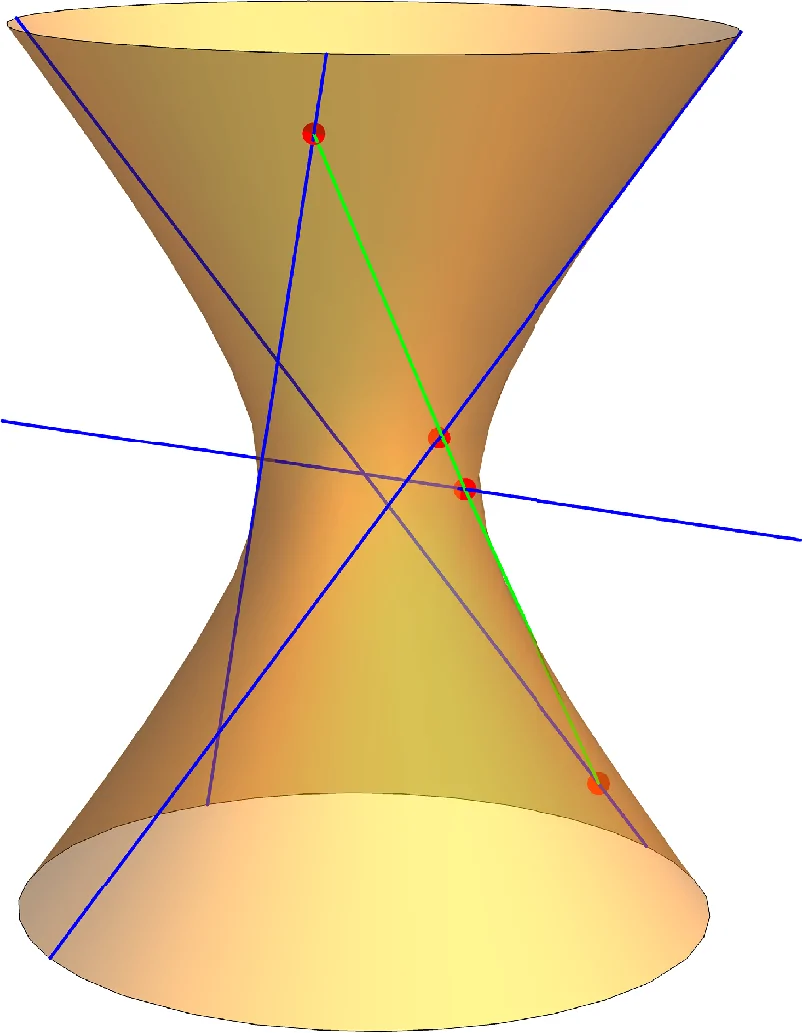
Three skew lines in a regulus, a fourth skew line which intersects the regulus twice, and a green transversal line. The four intersection points are shown in red

Let us name the four axial lines $l_1,l_2,l_3,l_4$. Of these four pairwise skew lines, the first three $l_1,l_2,l_3$ define a regulus - recall that this is the union of all lines which intersect all of $l_1,l_2,l_3$, and is a quadratic surface which we will call *R*. There are three possibilities for how the fourth line interacts with this regulus. Firstly, it may be tangential to *R*. Secondly, it could intersect *R* twice. Lastly, it may be contained in *R* (and is therefore in the same ruling family of the regulus as $l_1,l_2,l_3$). In any of these cases, there will exist a transversal line of all $l_1,l_2,l_3,l_4$, coming from the second ruling of *R*. A depiction of this line is shown in Fig. 1. The construction is now simple; let $l_5$ be a transversal line of all $l_1,l_2,l_3,l_4$. If we define $\pi _{l_5,l_i}$ to be the plane defined by the two lines $l_5$ and $l_i$ for i=1,2,3,4, and we define $\Pi _1,\Pi _2,\Pi _3,\Pi _4$ to be four sheaves of size *n* with axial lines $l_1,l_2,l_3,l_4$ such that $\pi _{l_5,l_i} \in \Pi _i$, we see that the line $l_5$ is in the intersection of the four sheaves, giving infinitely many four-rich points.

### Proof of Theorem 1

We now give the proof of Theorem 1.

#### Proof

We begin with the four sheaves of planes $\Pi _1,\Pi _2,\Pi _3, \Pi _4$. Let $l_1,l_2,l_3,l_4$ be the corresponding axial lines of these sheaves, which are assumed to be pairwise skew. It is well known that triples of skew lines are projectively equivalent; that is, we may apply a projective transformation π such that

$$\begin{aligned} \pi (l_1)&= Z(x,y) = \left\{ [0:0:z:w] \in {\mathbb {P}}^3 \right\} \\ \pi (l_2)&= Z(z,y-w) = \left\{ [x:y:0:y] \in {\mathbb {P}}^3 \right\} \\ \pi (l_3)&= Z(x-w,z-w) = \left\{ [x:y:x:x] \in {\mathbb {P}}^3 \right\} . \end{aligned}$$

We then rename so that $l_1,l_2,l_3,l_4$ refer to the image of the previous lines under π. Affinely, $l_1$ is given by x=y=0, $l_2$ is given by y=1,z=0, and $l_3$ is given by x=z=1. By using the projective transformations which fix the lines $l_1,l_2,l_3$, we can gain further information about the position of $l_4$. Consider the following projective transformations, given in matrix form by

$$M_{a,b,c}:= \begin{pmatrix} 1 &  b &  0 &  0 \\ a &  1+b-a-c &  0 &  0 \\ 0 &  b &  1+b &  -b \\ a &  b &  c &  1-a-c \end{pmatrix}$$

where $a,b,c \in {\mathbb {C}}$, and the determinant of the matrix is non-zero. These projective transformations all map the lines $l_1,l_2,l_3$ to themselves. The fourth line $l_4$ exists somewhere in ${\mathbb {P}}^3$, and we fix any affine plane (i.e. not the plane at infinity) which contains $l_4$, and does not contain either of the points [0 : 0 : 1 : 1], [0 : 0 : 0 : 1]. Note that such a choice always exists since $l_4$ is skew with the *z* axis $l_1$. We denote this plane by *H*, and write it as $t_1 x + t_2 y + t_3 z + t_4w =0$, for some $t_1,t_2,t_3,t_4 \in {\mathbb {C}}$. Since *H* does not contain the origin we have $t_4 \ne 0$, and so WLOG $t_4 = 1$, and we can write the points on this plane as

$$ H = \left\{ \left[ x:y:z:-t_1 x - t_2 y - t_3 z\right] : [x:y:z] \in {\mathbb {P}}^2\right\} .$$

We now consider the image of this plane under a transformation $M_{a,b,c}$, which are the points in ${\mathbb {P}}^3$ of the form

1 $$\begin{aligned} \begin{pmatrix} X \\ Y \\ Z \\ W \end{pmatrix} = \begin{pmatrix} b y+x \\ (b+1) y-y (a+c)+a x \\ b (t_1 x+y (t_2+1)+z (t_3+1))+z \\ b y+(a+c-1) (t_1 x+t_2 y+t_3 z)+a x+c z \end{pmatrix} \end{aligned}$$

for $[x:y:z] \in {\mathbb {P}}^2.$ Our aim will be to give a transformation $M_{a,b,c}$ such that $M_{a,b,c}(l_4)$ lies in the plane X+Y+Z=0 (note that we use capitals for these variables to distinguish them from the points parametrising *H*). This means that we need to find *a*, *b*, *c* such that for all $[x:y:z] \in {\mathbb {P}}^2$, the points in (1) satisfy X+Y+Z=0. We calculate that the sum of the first three coordinates above gives

$$(1+a+b t_1)x +(1+(3+t_2)b -a-c)y + (1+b(1+t_3))z.$$

This yields the solution

2 $$\begin{aligned} a = \frac{t_1-t_3-1}{t_3+1}, \quad b = \frac{-1}{t_3 + 1}, \quad c = \frac{-t_1-t_2+2t_3-1}{t_3+1}. \end{aligned}$$

Note that we cannot have $t_3= -1$, since this would imply that [0 : 0 : 1 : 1] lies on *H*, which we have assumed is not the case. Therefore this choice of *a*, *b*, *c* is valid. The determinant of the corresponding matrix $M_{a,b,c}$ is $\frac{(1+t_1+t_2+t_3+t_2t_3)^2}{(1+t_3)^4}$. We will now split into two cases, depending on whether there is some choice of $t_1,t_2,t_3$ such that $\frac{(1+t_1+t_2+t_3+t_2t_3)^2}{(1+t_3)^4} \ne 0$.

### Case 1

Assume that there exist $t_1,t_2,t_3$ as above, such that $\frac{(1+t_1+t_2+t_3+t_2t_3)^2}{(1+t_3)^4} \ne 0$. In this case, we find a projective transformation $M_{a,b,c}$ with *a*, *b*, *c* as given in (2), which maps the normalised $l_1,l_2,l_3$ onto themselves, and maps $l_4$ onto the plane X+Y+Z=0. We will now use Theorem 4 to bound the number of four-rich points defined by four sheaves of *n* planes, with the four axial lines $l_1,l_2,l_3,l_4$. Note that projective transformations map planes to planes and lines to lines, and will preserve the number of four rich points. Let us first assume that $l_4$ lies within the affine part of the plane X+Y+Z=0 (that is, $l_4$ does not lie entirely on the plane at infinity). In this case, we can write $l_4$ as the intersection of the plane X+Y+Z=0 and a second, vertical plane $s_1 X + s_2 Y +1 =0$. Note that we can assume the second plane does not pass through the origin.

Consider the first sheaf of planes, $\Pi _1$. These planes all contain the *z*-axis, and any such plane can be described as the zero set of the equation aX+bY=0, for some $[a:b] \in {\mathbb {P}}^1$. For our calculations, it will be more convenient to work with planes parametrised by a single complex number, as opposed to ${\mathbb {P}}^1$. To do this, we will remove the single plane Y=0 from $\Pi _1$ (if it is present in $\Pi _1$), allowing us to write planes in $\Pi _1$ as $X = m_1 Y$ for some complex number $m_1$. We claim that by removing any single plane from a sheaf, we are removing at most $O(n^2)$ four-rich points; indeed, for any plane $\pi \in \Pi _1$, the other three sheaves each intersect π to give three sets of *n* lines, call them $L_1,L_2,L_3$ contained in π. By our assumption on transversal lines, the intersection $L_1 \cap L_2 \cap L_3$ contains no lines, and so has size at most $O(n^2)$. These are precisely the four rich points contained in π, proving our claim.

Using the above argument, after removing at most one plane from each sheaf (and therefore removing at most $O(n^2)$ four-rich points, which is far smaller than our claimed bound), we can now write the four sheaves of planes as

$$\begin{aligned} \Pi _1 = \{ X=m_1 Y: m_1 \in M_1\} \\ \Pi _2 = \{ Y-1=m_2 Z: m_2 \in M_2\} \\ \Pi _3 = \{ Z-1=m_3 (X-1): m_3 \in M_3\} \\ \Pi _4 = \{ (s_1 + m_4)X + (s_2 + m_4)Y+ m_4 Z + 1=0: m_4 \in M_4\} \end{aligned}$$

where $M_1,M_2,M_3,M_4 \subseteq {\mathbb {C}}$ are finite sets of size *n*. Let *P* be the set of four rich points. Each point $(x_0,y_0,z_0)\in P$ yields a quadruple $(m_1,m_2,m_3,m_4) \in M_1 \times M_2 \times M_3 \times M_4$ such that the four equations

3 $$\begin{aligned} x_0=m_1 y_0 \end{aligned}$$

4 $$\begin{aligned} y_0-1=m_2 z_0 \end{aligned}$$

5 $$\begin{aligned} z_0-1=m_3 (x_0-1) \end{aligned}$$

6 $$\begin{aligned} (s_1 + m_4)x_0 + (s_2 + m_4)y_0+ m_4 z_0 + 1=0 \end{aligned}$$

are satisfied. Eliminating $x_0,y_0,z_0$ from this system yields the polynomial equation $F(m_1,m_2,m_3,m_4)=0$, where

$$\begin{aligned}  &   F(x,y,z,w):=z(xy (w+s_1+1)-xw+y (w+s_2)+w)\\  &   \quad -w (x y+x+y+2)+x (y+1) s_1+y s_2+s_2+1. \end{aligned}$$

Now consider the projection map

**Figure:**
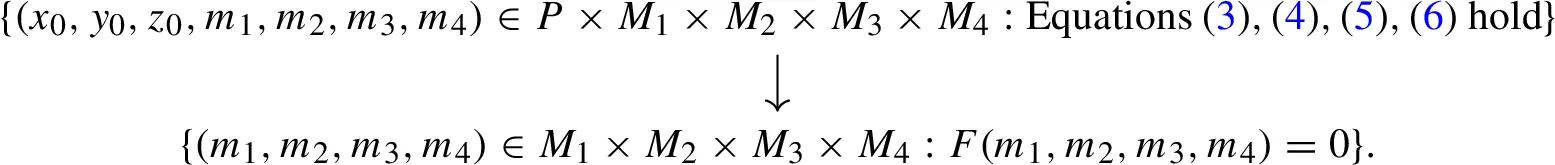

We claim that as long as no transversal line *l* of $l_1,l_2,l_3,l_4$ is contained in a plane from each of the $\Pi _i$, this projection is a bijection. Indeed, given four planes corresponding to $(m_1,m_2,m_3,m_4)$ in the image set of the projection, these four planes either intersect in a single point, or they intersect in a full line (note that two planes cannot be equal since their axial lines are skew). However we see that if the four planes intersect in a line, then that line is a transversal line to $l_1,l_2,l_3,l_4$. Since we have assumed that this does not happen, the projection above is a bijection. We now aim to upper bound the size of the image set of this projection.

#### Irreducibility of *F*

In order to prove that *F* is irreducible, we will use a well know result concerning polynomial resultants. For a proof of the following result, see [1, Section 3.6, Proposition 1]. We will freely use this lemma throughout the paper.

##### Lemma 3

Suppose that $f,g\in {\mathbb {C}}[x,y,z,w]$ are polynomials which have a non-trivial common factor that depends on *x*. Then the resultant $\text {Res}(f,g,x)$ is identically zero.

We shall now prove that *F* is irreducible for all possible values of $s_1,s_2$. Indeed, let us suppose that *F* is reducible, so that there exist $g,h \in {\mathbb {C}}[x,y,z,w]$ non-trivial such that F=gh. *F* itself has a term -2w, and so WLOG we may assume that *g* has a term of the form λw for some $\lambda \ne 0$. Furthermore, *F* has no term with a factor of $w^2$. This implies that *h* has no *w* variable appearing at all, that is, $h \in \mathbb {C}[x,y,z]$.

Now consider the partial derivative $F_w$. We have F=gh, and so $F_w = g_w h + g h_w = g_w h$, since $h_w =0$. Therefore *F* and $F_w$ have the non-trivial common factor $h\in \mathbb {C}[x,y,z]$. For any of the variables *x*, *y*, *z*, we can consider *F* and $F_w$ as univariate polynomials in that variable. Therefore since *F* and $F_w$ have the common factor *h* which depends on at least one of these variables, at least one of the resultants $\text {Res}(F,F_w,x),\text {Res}(F,F_w,y),\text {Res}(F,F_w,z)$ must be identically zero. Calculating these resultants with Mathematica, we find

$$\begin{aligned} \text {Res}(F,F_w,x) = (yz-y-1) (s_1 (y (z-1)+z-2)+s_2 (1+y+z-yz)+y z+z+1) \\ \text {Res}(F,F_w,y) = (z-xz-1) (1 + s_2 (z-1) + x (1 + s_1 (z-1) + z)) \\ \text {Res}(F,F_w,z) = (1 - x - x y) (1 + s_2 + y + x (s_1 + y)). \end{aligned}$$

For any $s_1,s_2$, all of these resultants are non-zero - this can be easily checked by considering coefficients. For instance, if $\text {Res}(F,F_w,z)$ is identically zero, we must have that its second factor is zero. However this second factor always has a term *xy*, and so can never be zero. Similar arguments hold for the other resultants. Therefore, *F* is an irreducible polynomial for all values of $s_1,s_2$.

#### Applying Elekes-Szabó

We now need to check whether the irreducible polynomial *F*(*x*, *y*, *z*, *w*) is a degenerate polynomial. On the non-trivial Zariski-open (and therefore Euclidean open) subset of ${\mathbb {C}}^3$ given by $(x y (w+s_1+1)-x w+y (w+s_2)+w) \ne 0$, we can write *Z*(*F*) as the graph of the function z=f(x,y,w) where

$$f(x,y,w):= \frac{-w (x y+x+y+2)+x (y+1) s_1+y s_2+s_2+1}{x y (w+s_1+1)-x w+y (w+s_2)+w}.$$

We can now use Lemma 2. Using Mathematica, we calculate that the derivative $\frac{\partial }{\partial x}\frac{f_y}{f_w}$ is given by the following expression.

**Figure:**
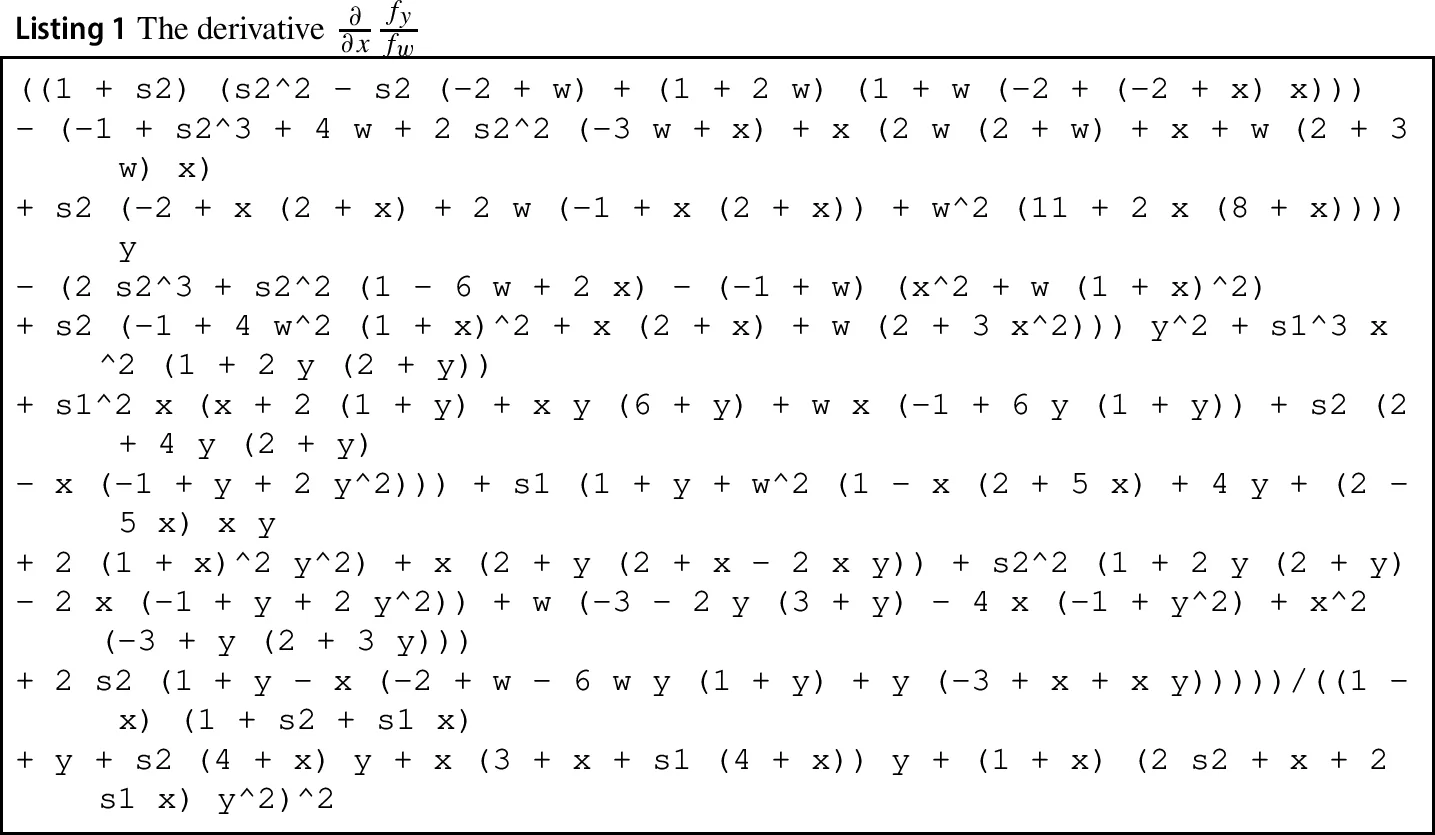

Using Mathematica, this function is verified to not vanish on any non-trivial open set. Therefore by Lemma 2 the polynomial *F* is non-degenerate. Recalling that *P* denotes the set of four rich points, we then find

$$|P| \le |Z(F) \cap M_1 \times M_2 \times M_3 \times M_4| \ll n^{8/3}$$

as needed.

#### When $l_4$ Lies on the Plane at Infinity

We now deal with the case where $l_4$ is equal to the line at infinity of the plane X+Y+Z=0. If this is the case, the sheaf of planes $\Pi _4$ can be written as (excluding the single plane at infinity, since any single plane can contain only $O(n^2)$ four-rich points under our assumptions)

$$\Pi _4 = \{X+Y+Z=m_4:\ m_4 \in M_4\}$$

for some finite set $M_4 \subseteq {\mathbb {C}}$. Therefore any four rich point $(x_0,y_0,z_0)$ again gives a solution $(m_1,m_2,m_3,m_4) \in M_1\times M_2\times M_3 \times M_4$ to the three equations (3),(4),(5), and the equation

$$x_0+y_0+z_0 = m_4.$$

Following the same procedure as above, we eliminate these four equations down to the single equation

$$F(m_1,m_2,m_3,m_4)=2 + m_2 + m_1 (1 + m_2 + m_3 + m_2 m_3 (m_4 -1)) - (m_3 + m_2 m_3 + m_4)=0.$$

This polynomial *F* is irreducible, and can be verified as non-degenerate using Lemma 2. We do not give the details as the method is identical to that above. Therefore in this case we also find $O(n^{8/3})$ four-rich points.

### Case 2

We now consider the case where all planes $t_1 x + t_2 y + t_3 z + 1$ containing $l_4$ satisfy $1+t_1+t_2+t_3+t_2t_3 = 0$, that is, the corresponding matrix $M_{a,b,c}$ with *a*, *b*, *c* as in (2) has determinant zero. We claim that if this is the case, then $l_4$ lies in a common regulus with $l_1,l_2,l_3$. To prove this claim, we use point-plane duality to swap the sheaf of planes containing $l_4$ for a line, call it *L*, in ${\mathbb {P}}^3$; specifically the line of points $[t_1:t_2:t_3:1]$ such that the corresponding plane $t_1 x + t_2 y + t_3 z + 1$ contains $l_4$. Note that as we removed the plane containing $l_4$ and the origin, we are missing the one point at infinity of this line. By assumption, this line lies within a doubly ruled surface *R* given by the equation 1+X+Y+Z+YZ=0 (in this section we shall use capital letters to distinguish the dual space from the primal space). The equation defining *R* can be written as X=-(1+Y)(1+Z), and the two ruling families of lines within *R* can be written as

$$\begin{aligned} {\mathcal {L}}_1:= \{l_{\alpha }:\alpha \in {\mathbb {C}}\},\quad l_{\alpha }:= \{(X,Y,Z): -(1+Y)=\alpha , \ \alpha (1+Z) = X \} \\ {\mathcal {L}}_2:= \{l_{\beta }':\beta \in {\mathbb {C}}\},\quad l_{\beta }':= \{(X,Y,Z): (1+Z)=\beta , \ -\beta (1+Y) = X \} \end{aligned}$$

We claim that the line *L* must lie within the family ${\mathcal {L}}_1$. Indeed, suppose it lies in ${\mathcal {L}}_2$. Then for some $\beta \in {\mathbb {C}}$, the sheaf of all planes containing $l_4$ are given by equations of the form

$$-\beta (1+s)x + sy +(\beta -1)z + 1=0, \quad s \in {\mathbb {C}}.$$

However, all of these planes pass through the point $\left( 0,0,\frac{1}{1-\beta }\right)$, implying that $l_4$ must also contain this point, contradicting the skewness of $l_1$ and $l_4$ (recall that after normalisation $l_1$ is the *z*-axis). If we have that β=1, the resulting planes all pass through the point [0 : 0 : 1 : 0], yielding the same contradiction. Therefore $L \in {\mathcal {L}}_1$.

Therefore, for some $\alpha \in {\mathbb {C}}$ the sheaf of all planes containing $l_4$ are of the form

7 $$\begin{aligned} \alpha (1+s)x - (1+\alpha )y + sz + 1=0, \ \quad s \in {\mathbb {C}}. \end{aligned}$$

Excluding the case α=-1, the axial line $l_4$ of this sheaf is the set of points

$$\left( t,\frac{\alpha t +1}{\alpha + 1},-\alpha t\right) , \quad t \in {\mathbb {C}}.$$

The case α=-1 corresponds to the axial line $l_4$ being equal to the set of points (1, *y*, 1) for $y \in {\mathbb {C}}$, which is the axial line $l_3$ - therefore this case does not happen since $l_3$ and $l_4$ are skew. We now see that all axial lines $l_1,l_2,l_3,l_4$ lie within the regulus given by the equation xy+xz-yz-x=0. Furthermore, the case α=0 can also be excluded, as in this case $l_4$ would be the same as $l_2$. We can now continue with the proof as in Case 1. The first three sheaves of planes are the same $\Pi _1,\Pi _2,\Pi _3$ from Case 1, with corresponding sets $M_1,M_2,M_3 \subseteq {\mathbb {C}}$. The final sheaf now consists of planes of the form (7), with $s \in M_4$ for some finite set $M_4 \subseteq {\mathbb {C}}$. As in Case 1, any four-rich point $(x_0,y_0,z_0) \in P$ yields a quadruple $(m_1,m_2,m_3,m_4) \in M_1 \times M_2 \times M_3 \times M_4$ such that the equations

$$\begin{aligned} x_0=m_1 y_0 \\ y_0-1=m_2 z_0 \\ z_0-1=m_3 (x_0-1) \\ \alpha (1+m_4)x_0 - (1+\alpha )y_0 + m_4 z_0 + 1=0. \end{aligned}$$

We will again attempt to bound the number of tuples $(x_0,y_0,z_0,m_1,m_2,m_3,m_4)$ by eliminating these four equations down to one equation in $(m_1,m_2,m_3,m_4)$. For this to be valid, we again need the condition that no transversal line of $l_1,l_2,l_3,l_4$ is contained in a plane from each sheaf, so that the analogous projection of that used in Case 1 is also a bijection. If this is the case, we proceed to eliminate down to a single polynomial equation $F(m_1,m_2,m_3,m_4) = 0$, for $F \in {\mathbb {C}}[x,y,z,w]$. This polynomial is given by

F(x,y,z,w)=α(xw+x-1)(y(z-1)-1)+((x-1)z+1)(y-w)

#### Irreducibility of *F*

We will use the same method as in Sect. 3.3.1 to prove that *F* is irreducible for all $\alpha \in {\mathbb {C}} \setminus \{-1,0\}$. As we have seen, these two excluded values of α only occur when $l_4 = l_3$ or $l_4 = l_2$, which is not the case.

Assume that *F* were reducible, so that F=gh for some non-trivial $g,h \in {\mathbb {C}}[x,y,z,w]$. *F* itself has a term -w, and so WLOG we can assume that *g* has a term of the form λw for some $\lambda \ne 0$. Using the same proof as in Sect. 3.3.1, we can see that *h* must then not contain any occurrence of the variable *w*, so that $h_w = 0$, and therefore we again have that *F* and $F_w$ have the common factor *h*. Again considering *F* as univariate in the variables *x*, *y*, *z* in turn, we must have that one of the following resultants is zero.

$$\begin{aligned} \text {Res}(F,F_w,x) = \alpha (1 + \alpha ) (1 + y - y z)^2 \\ \text {Res}(F,F_w,y) = -(1 + \alpha ) (1 + (x-1) z)^2 \\ \text {Res}(F,F_w,z) = -\alpha (xy+x-1)^2. \end{aligned}$$

We now see that as long as $\alpha \ne 0,-1$, all of these resultants are non-zero. Therefore *F* is irreducible for all valid choices of α.

#### Applying Elekes-Szabo

We shall now use Lemma 2 to prove that *F* is non-degenerate. Indeed, on the Zariski-open set ${\mathbb {C}}^3 \setminus Z(\alpha (1 + w) ( y (z-1)-1) + (y-w) z)$, the hypersurface *Z*(*F*) can be written as the graph of the function

$$x = f(y,z,w) = \frac{w - y - \alpha (1 + y) - w z + (1 + \alpha ) y z}{\alpha (1 + w) ( y (z-1)-1) + (y-w) z}.$$

We then calculate

$$\frac{\partial }{\partial y}\left( \frac{f_z}{f_w}\right) = \frac{2 (w-y) (w (z-1)-1)}{\alpha (y (z-1)-1)^3}$$

which does not vanish on any open set (recall that $\alpha \ne 0$). Therefore *F* is non-degenerate. Applying Theorem 4 then shows that the number of four rich points is at most $O(n^{8/3})$, as required. This concludes the proof of Theorem 1. □

## Rich Points from Concentric Spheres

In this section we prove Theorem 2, which we restate here for convenience.

### Theorem

Let ${\mathcal {S}}_1, {\mathcal {S}}_2, {\mathcal {S}}_3, {\mathcal {S}}_4$ be four sets of *n* concentric spheres in ${\mathbb {C}}^3$, whose centres do not lie on a common plane, and no three centres are collinear. Then the number of four rich points determined by these four sets is $O(n^{8/3})$.

Before proving this result, we give a construction showing that an assumption on the centres of the sets of spheres in necessary.

### Construction

We give the following construction of four points which each define only $|P|^{1/3}$ distances to some point set *P*. Note that the construction is equivalent to giving four pencils of *n* spheres, with three centres collinear, which define $n^3$ four-rich points. We note that if all four centres of the spheres are collinear, one can also give a construction giving infinitely many four-rich points (for instance, by having a sphere from each family contain a fixed circle).

#### Construction 2

There exists a set of points $q_1,q_2,q_3,q_4 \in {\mathbb {R}}^3$, with $q_1,q_2,q_3$ on a common line, and four sets of *n* concentric spheres ${\mathcal {S}}_1,{\mathcal {S}}_2, {\mathcal {S}}_3, {\mathcal {S}}_4$, with ${\mathcal {S}}_i$ centred around $q_i$, which determine $\Omega (n^3)$ four rich points.

#### Proof

The construction begins in two dimensions; a construction of Elekes [3] gives three collinear points in the plane ${\mathbb {R}}^2 \subseteq {\mathbb {C}}^2$, together with *n* circles around each point, such that there are $\Omega (n^2)$ three-rich points. Indeed, the three points can be taken as (-1,0),(0,0), and (1, 0). The circles around each point are defined to have radii $\sqrt{i}$, for i∈[n].

We now extend this construction to $\mathbb {R}^3$, by giving each point a third coordinate with value 0, and by defining spheres instead of circles (with the same radii as above). These three points are $q_1,q_2,q_3$, and the three sets of spheres are ${\mathcal {S}}_1,{\mathcal {S}}_2, {\mathcal {S}}_3$. Note that intersecting this construction with any plane containing the *x*-axis yields Elekes’ construction in the plane. The three families of *n* spheres yield $\Omega (n^2)$ three-rich *circles*, which can be seen as any triple point given by Elekes’ construction can be fully rotated around the *x*-axis. We can now choose $q_4$ to be placed on the *y*-axis, very far from the origin. By placing *n* spheres ${\mathcal {S}}_4$ around $q_4$, each sphere intersects each of the $\Omega (n^2)$ three-rich circles, as long as $q_4$ is far enough away and the radii of the spheres are very close to $||q_4||$. This yields a set of $\Omega (n^3)$ four rich points. □

### Proof of Theorem 2

We now prove Theorem 2.

#### Proof

Let ${\mathcal {S}}_1, {\mathcal {S}}_2, {\mathcal {S}}_3, {\mathcal {S}}_4$ be the four sets of concentric spheres, each of size *n*. By translating, we can assume that ${\mathcal {S}}_1$ is centred at the origin. We can also rotate and dilate so that ${\mathcal {S}}_2$ is centred at (1, 0, 0). Finally, by rotating around the *x*-axis, we can assume that the spheres from the third set ${\mathcal {S}}_3$ are centred at some point (*a*, *b*, 0), with b≠0 since no three centres are collinear. The final set ${\mathcal {S}}_4$ is now centred at some point (*c*, *d*, *e*) with e≠0 since the four points are not coplanar.

Suppose that (*x*, *y*, *z*) is a four-rich point. Let us denote the set of such four-rich points by $P_4$. By identifying a sphere from each family by its squared radius (that is, each set ${\mathcal {S}}_i$ has a corresponding set $R_i \subseteq {\mathbb {C}}$ of its squared radii), we obtain a solution $(x,y,z,t_1,t_2,t_3,t_4) \in P_4 \times R_1 \times R_2 \times R_3 \times R_4$ to the equations

$$\begin{aligned} t_1&= x^2 + y^2 + z^2 \\ t_2&= (x-1)^2 + y^2 + z^2 \\ t_3&= (x-a)^2 + (y-b)^2 + z^2 \\ t_4&= (x-c)^2 + (y-d)^2 + (z-e)^2. \end{aligned}$$

Let *A* be the set of these solutions. Using Mathematica, we can eliminate *x*, *y*, *z* from these equations, to obtain a single polynomial equation $F(t_1,t_2,t_3,t_4)=0$. The polynomial $F(t_1,t_2,t_3,t_4)$ is shown below. Let *B* be the set of solutions to $F(t_1,t_2,t_3,t_4) =0$ within $R_1\times R_2 \times R_3 \times R_4$.

**Figure:**
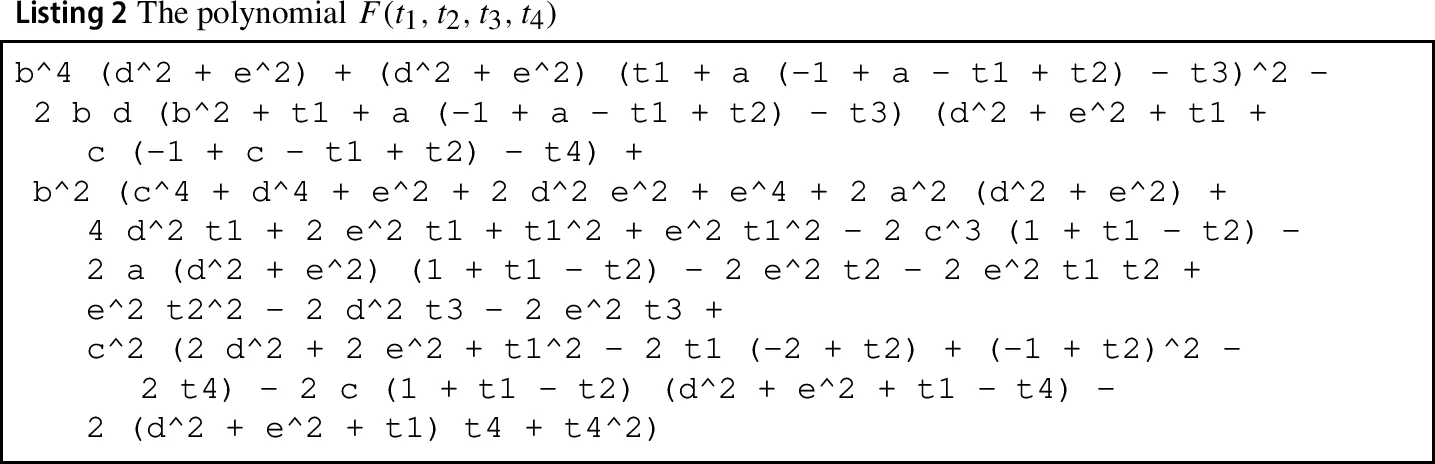

As in the proof of Theorem 1, we have a corresponding map $\pi :A \rightarrow B$, which is well defined by the reduction above. We claim that any element in the image of π has one preimage. Indeed, given the quadruple $(t_1,t_2,t_3,t_4)$, the four sphere equations can be used to uniquely determine (*x*, *y*, *z*), using our assumptions that b,e≠0, and because for instance when the first equation is taken away from the second, we obtain a linear equation in *x*. This is then repeated to obtain a linear equation in each of *x*, *y*, *z*, giving a unique pre-image.

### Irreducibility of *F*

We shall now prove that *F* is irreducible. Assuming that *F* is in fact reducible, we have that F=gh with *g*, *h* non-constant in ${\mathbb {C}}[t_1,t_2,t_3,t_4]$. Since the total degree of *F* is two, *g* and *h* must be linear polynomials. We then must have

$$F = (g_1 t_1 + g_2 t_2 + g_3 t_3 + g_4 t_4 + g_5)(h_1 t_1 + h_2 t_2 + h_3 t_3 + h_4 t_4 + h_5)$$

for some $g_1,...,g_5,h_1,...,h_5 \in {\mathbb {C}}$. Using this form as an Ansatz, we can compare coefficients using Mathematica to find that *F* is reducible if and only if be=0, which is not the case for us. Therefore *F* is irreducible.

### Application of Elekes-Szabó

We now rewrite $F(t_1,t_2,t_3,t_4)=0$ as $t_4 = f(t_1,t_2,t_3)$ for $(t_1,t_2,t_3)$ on some non-empty Zariski open set. Making use of Mathematica, we can calculate the expression $\frac{\partial }{\partial t_1} \left( \frac{f_{t_2}}{f_{t_3}}\right)$ - it is too long to state here, but can be calculated using the code in the appendix. For b,e≠0, this expression does not vanish on any open set, and therefore $F(t_1,t_2,t_3,t_4)$ is non-degenerate by Lemma 2. By Theorem 4, we then have

$$|P_4| \le |Z(F) \cap R_1\times R_2 \times R_3 \times R_4| \ll n^{8/3}$$

which concludes the proof. □

### Pinned Distance Corollary

Corollary 1 can now be easily deduced from Theorem 2. Indeed, given the finite set *P*, we can cover *P* with spheres around each $q_1,q_2,q_3,q_4$. One of these covering sets of spheres has the largest size; we add arbitrary spheres to the other sets until they all have this maximum size. Since *P* is a subset of the four-rich points defined by these spheres, Theorem 2 then implies

$$|P| \ll \max _i(|D(q_i,P)|)^{8/3}$$

and the result follows.

Note that it would not be hard to follow the proof of Theorem 2 with the sets of spheres being of different sizes, to obtain an ‘unbalanced’ version. We do not pursue this here, in order to simplify the calculations.

## Pencils in ${\mathbb {R}}^2$

In this section we prove an upper bound on the number of four rich points determined by two pencils of lines and two pencils of concentric circles within $\mathbb {R}^2$. This follows the work on four-rich points determined by four pencils of *n* lines in [8]. Roche-Newton and Warren proved the upper bound $O(n^{11/6}),$ which, even though it matches the previous best Elekes-Szabó bound [7], relies on techniques different to ours and cannot be improved with the method demonstrated in this paper. Assuming all the pencils have size *n*, we will use the three-dimensional Elekes-Szabó Theorem (Theorem 3) to obtain an upper bound of $O(n^{12/7})$. We note that using the same proof as below, one can prove many variants, concerning the number of four-rich points defined by different configurations of circles and lines - we choose to prove this particular variant as it is relatively simple while still showing the general method. Furthermore, one may extend these results to $\mathbb {C}^2$ using the complex variant of Theorem 3, found in [5], at a cost of the weaker bound $O(n^{11/6})$. We prove the following result.

### Theorem 5

Let $\mathcal {C}_1,\mathcal {C}_2$ be two pencils of *n* concentric circles with distinct centres and non-zero radii, and $\mathcal {L}_1,\mathcal {L}_2$ be two pencils of *n* concurrent lines with distinct centres in the plane $\mathbb {R}^2$. Then the number of four-rich points $O(n^{12/7})$.

**Figure:**
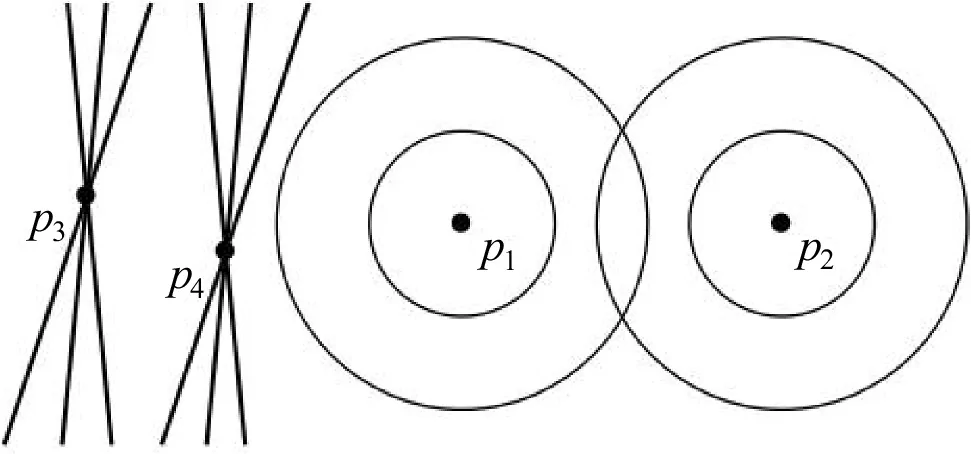


### Proof

Identify each circle of $\mathcal {C}_1,\mathcal {C}_2$ with its square radius, coming from corresponding sets $R_1,R_2\subseteq \mathbb {R}^*$ respectively, and each line of $\mathcal {L}_1,\mathcal {L}_2$ with its slope, coming from the sets $S_1,S_2\subseteq \mathbb {R}$, where all sets have size *n*. We remove any vertical lines, since any single line has at most 2*n* four rich points lying on it, far fewer than we claim. Furthermore, if the line connecting $p_1$ and $p_2$ is in either pencil of lines, we remove it also. Without loss of generality, by rotations, translations and dilations we can assume that the circles in $\mathcal {C}_1$ are centred at the origin $p_1=(0,0)$ and those in $\mathcal {C}_2$ have centre $p_2=(1,0)$, and let the centres of $\mathcal {L}_1,\mathcal {L}_2$ be at some points $p_3=(a,b),\;p_4=(c,d)$. Let $P_4$ be the set of four rich points. For each four rich point $(x,y) \in P_4$, there exist $(t_1,t_2,t_3,t_4) \in R_1 \times R_2 \times S_1 \times S_2$ such that we have a solution $(x,y,t_1,t_2,t_3,t_4)\in P_4\times R_1\times R_2\times S_1\times S_2$ to the equations

$$\begin{aligned} x^2+y^2=t_1\\ (x-1)^2+y^2=t_2\\ y-b=t_3(x-a)\\ y-d=t_4(x-c). \end{aligned}$$

Let us call the set of these solutions *A*. Using Mathematica, we can eliminate $x,y,t_2$ from this system of equations and obtain a single expression $F(t_1,t_3,t_4)=0,$ where *F* is the polynomial

$$\begin{aligned} t_3^2 \left( a^2+d^2\right) +t_4^2 \left( (t_3 (c-a)+b)^2+c^2-t_1\right) +2 t_3 t_4 (a d t_3+t_1)\\+2 a d t_3+b^2+d^2-2 b (a t_3-c t_4+d t_3 t_4+d)-2 c t_4 \left( a t_3+d t_3^2+d\right) -t_1 t_3^2. \end{aligned}$$

Now let *B* be the set of solutions $(t_1,t_3,t_4) \in R_1 \times S_1 \times S_2$ to $F(t_1,t_3,t_4)=0$, that is,

$$B = Z(F) \cap R_1 \times S_1 \times S_2.$$

The above reduction shows that we have a map $\pi : A \rightarrow B$ given by $\pi (x,y,t_1,t_2,t_3,t_4)= (t_1,t_3,t_4)$.

### Claim 1

For each $(t_1,t_3,t_4) \in \pi (A) \subseteq B$, there is a unique pre-image under π.

### Proof of Claim

Firstly note that if $t_3 = t_4$, then the two lines through $p_3$ and $p_4$ are parallel. They cannot be the same line, since we removed this line if it was present. Therefore the two lines do not intersect, and we find no pre-image.

Now suppose that $t_3 \ne t_4$, so that the two lines through $p_3$ and $p_4$ are not parallel. They therefore have a unique intersection point (*x*, *y*), which must be the four-rich point in the preimage. Wherever $p_2$ lies, the entry $t_2$ in the preimage must be the squared distance between (*x*, *y*) and $p_2$, which is uniquely determined by (*x*, *y*). Therefore we find a unique preimage. □

Given the claim, we have that $|P_4| \le |A| \le |B|$, and so we now aim to upper bound |*B*| by applying Theorem 3 to *F*.

We check the irreducibility of *F* using the method demonstrated in Sect. 3.3.1. Assume for contradiction that F=gh for some non-constant polynomials $g,h\in \mathbb {R}[t_1,t_3,t_4].$ Observe that a term involving $t_1$ appears in *F*, but no term involves $t_1^2.$ Hence, without loss of generality, suppose that *g* contains a non-zero term involving $t_1$, while *h* does not. The partial derivative of *F* with respect to $t_1,$ $F_{t_1}=g_{t_1}h$, has the common factor *h* with *F*. Consequently, at least one of $Res(F,F_{t_1},t_3)$ and $Res(F,F_{t_1},t_4)$ must be zero. Using Mathematica, we obtain

$$\begin{aligned} Res(F,F_{t_1},t_3)=(b - d + (c-a) t_4)^4 (1 + t_4^2)^2\\ Res(F,F_{t_1},t_4)=(b - d + (c-a) t_3)^4 (1 + t_3^2)^2. \end{aligned}$$

Both resultants are zero precisely when a=c and b=d, which cannot happen due to our condition that the centres of $\mathcal {L}_1$ and $\mathcal {L}_2$ are distinct, yielding that *F* is irreducible.Finally, solving F=0 with respect to $t_1$ yields the equation

$$\begin{aligned} t_1=\frac{1}{(t_3-t_4)^2}\Big (a^2 t_3^2t_4^2+a^2 t_3^2-2 a b t_3 t_4^2-2 a b t_3-2 a c t_3^2 t_4^2-2 a c t_3 t_4+2 a dt_3^2 t_4\\+2 a d t_3+b^2 t_4^2+b^2+2 b c t_3 t_4^2+2 b c t_4-2 b d t_3 t_4-2 b d\\+c^2 t_3^2 t_4^2+c^2 t_4^2-2 c d t_3^2 t_4-2 c d t_4+d^2t_3^2+d^2\Big ), \end{aligned}$$

where on the right side we have a rational function to which we apply Lemma 1. Under the condition that (a,b)≠(c,d) the resulting expression does not vanish on any open set, so *F* is not degenerate. By Theorem 3 we conclude that

$$\begin{aligned} |P_4| \le |A| \le |B| \le 2|V(F)\cap R_1\times S_1\times S_2|\ll n^{12/7}. \end{aligned}$$

□

## Data Availability

Data sharing is not applicable to this article as no datasets were generated or analysed during the current study.

## Appendix - Mathematica Code

Here we give a short Mathematica package which can be used to apply the 4-d degeneracy test.
